# Supplementary material for: A zero-valent palladium cluster-organic framework
Source: Nat Commun. 2024 Feb 8;15:1177. doi: 10.1038/s41467-024-45363-3 (PMC10853280; doi:10.1038/s41467-024-45363-3)
Supplement: Supplementary file 1 — Supplementary Information [file 41467_2024_45363_MOESM1_ESM.pdf]

Supplementary Information

A zero-valent palladium cluster-organic framework

Xiyue Liu,[1] James N. McPherson,[1] Carl Emil Andersen,[1] Mike S. B. Jørgensen,[1] René Wugt Larsen,[1] Nathan J. Yutronkie,[2] Fabrice Wilhelm,[2] Andrei Rogalev,[2] Mónica Giménez-Marqués,[3] Guillermo Mínguez Espallargas,[3] Christian R. Göb,[4] Kasper S. Pedersen[1]

[1]Department of Chemistry, Technical University of Denmark, Kemitrovet 207, 2800 Kgs. Lyngby, Denmark.

[2]European Synchrotron Radiation Facility (ESRF), CS 40220, 38043, Grenoble Cedex 9, France.

[3]Instituto de Ciencia Molecular (ICMol), Universidad de Valencia, Paterna 46980, Valencia, Spain.

[4]Rigaku Europe SE, Hugenottenallee 167, 63263 Neu-Isenburg, Germany.

Table of Contents

Supplementary Note 1 3
Supplementary Note 2 11
Supplementary Note 3 12
Supplementary Note 4 13
Supplementary Note 5 15
Supplementary Note 6 16
Supplementary Note 7 17
Supplementary Note 8 19
Supplementary Note 9 20
Supplementary Note 10 21
Supplementary References 24

Supplementary Note 1

Crystallography

Single-crystal X-ray diffraction

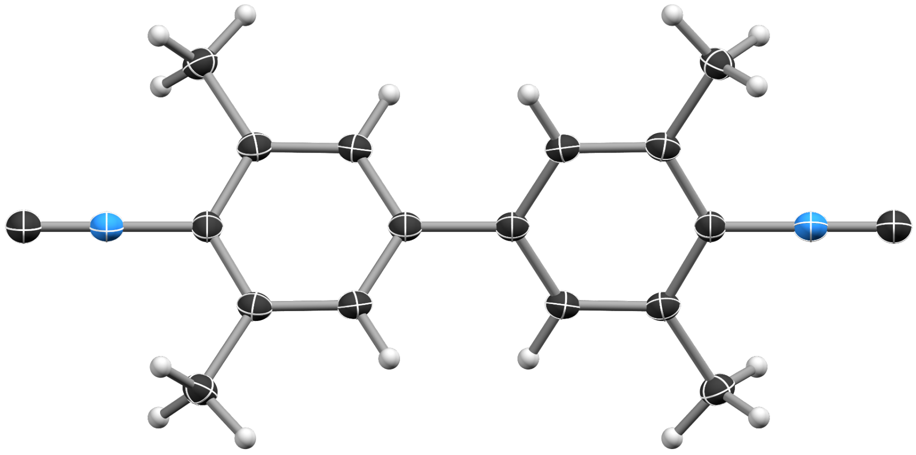

Supplementary Figure 1. An ORTEP diagram from the single-crystal X-ray diffraction structure of BXylDI, viewed down the crystallographic *a* axis showing 50% thermal ellipsoids at 120 K. Key bond distances (Å) and angles (°): C_{CN}≡N 1.1561(19); C_{CN}–N–C_{ipso} 180.

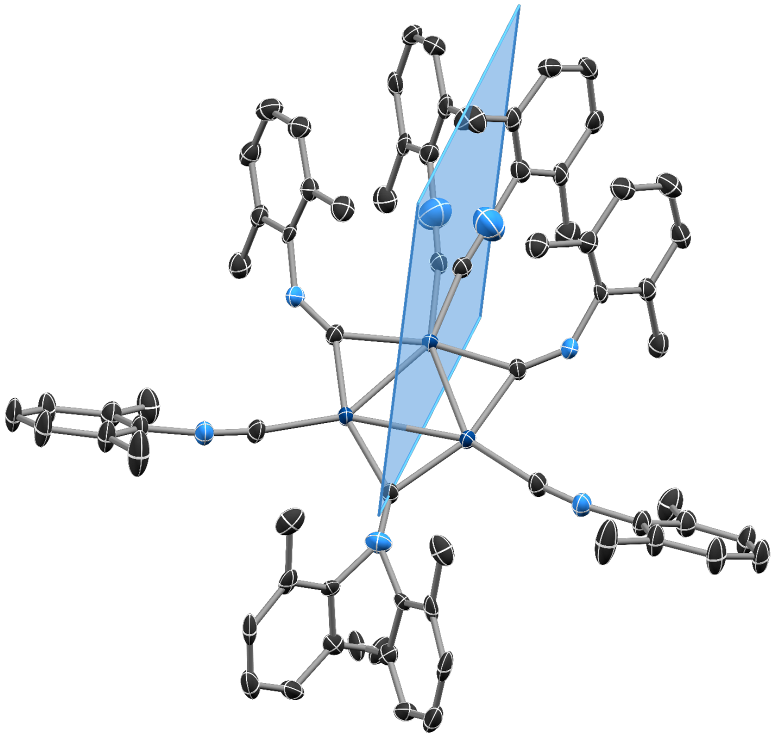

Supplementary Figure 2. ORTEP diagram from the single-crystal X-ray diffraction structure of $\mathbf{Pd_3}$, showing 50% thermal ellipsoids at 120 K, showing positional disorder across the (020) mirror plane bisecting the $\mathbf{Pd_3}$ molecule (blue plane). Hydrogen atoms have been omitted for clarity.

Continuous rotation 3D electron diffraction

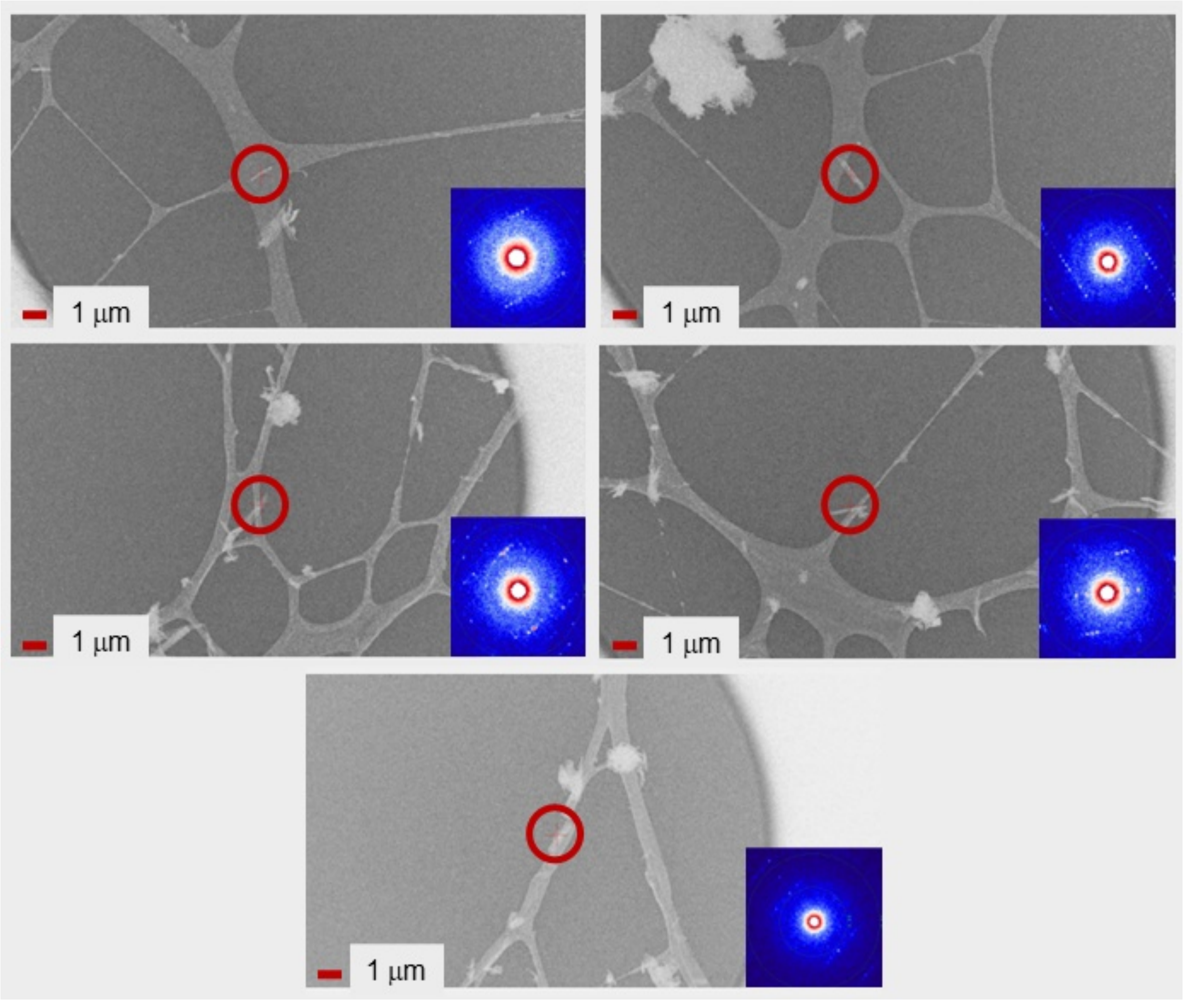

Supplementary Figure 3. Transmission Electron Microscope images alongside a single frame collected in diffraction mode of the five **Pd_3-MOF** crystals analysed by 3D-ED.

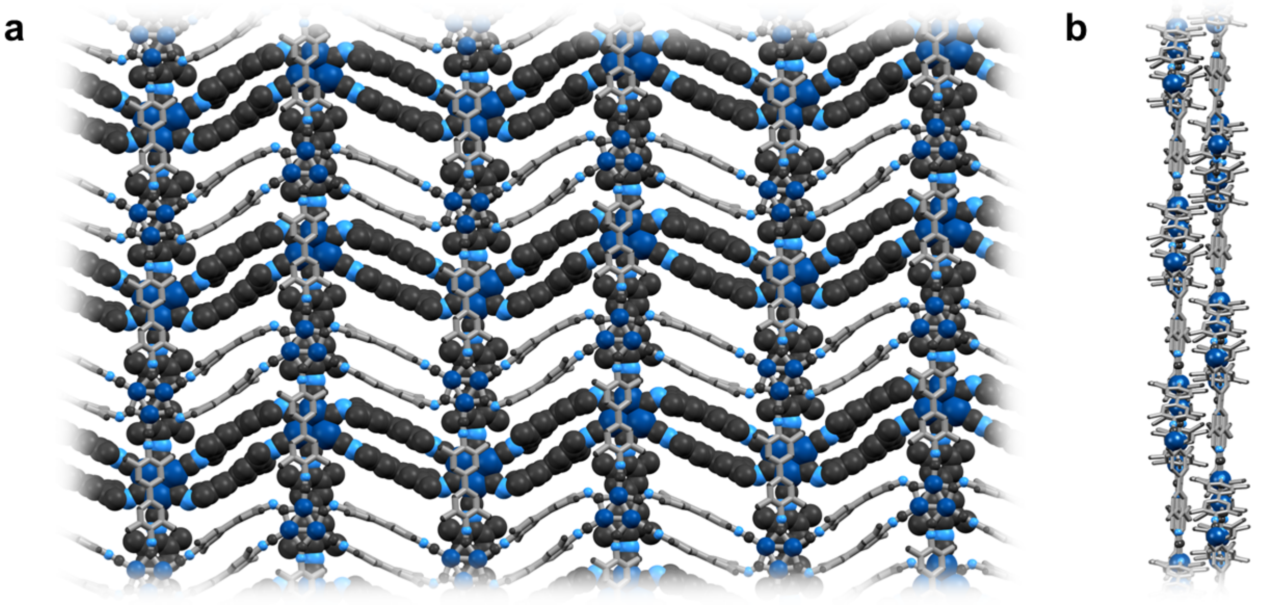

Supplementary Figure 4. Views of the 3D-ED structure of **Pd_3-MOF** showing two parallel, adjacent sheets orthogonal to (**a**) or along (**b**) the mean plane through one of the sheets. In view (**a**), the rear sheet is represented with spacefill balls.

Crystallographic Tables

Supplementary Table 1. SC-XRD data for BXylDI and **Pd_3**.

	**BXylDI**	**Pd_3**
CCDC Code	2293023	2293024
Empirical formula	$C_{18}H_{16}N_2$	$Pd_3(C_9H_9N)_6$
Formula weight	260.33	1120.24
Temperature (K)	120.0(1)	120.0(1)
Crystal system	Orthorhombic	Monoclinic
Space group	*Fddd*	*Cm*
a (Å)	7.4395(1)	7.5739(1)
b (Å)	16.8661(3)	25.84180(3)
c (Å)	22.9543(4)	11.8418(1)
α (°)	90	90
β (°)	90	93.326(1)
γ (°)	90	90
Volume (Å^3)	2880.20(8)	2314.52(5)
Z	8	2
ρ_{calc} (g cm^{-3})	1.201	1.607
μ (mm^{-1})	0.548	9.652
F(000)	1104.0	1130.0
Crystal size (mm^3)	0.302 × 0.119 × 0.064	0.156 × 0.07 × 0.051
Radiation	Cu Kα (λ = 1.5406 Å)	Cu Kα (λ = 1.5406 Å)
2 θ range (°)	13.028 to 153.512	6.84 to 152.802
Index ranges	$-8 \le h \le 9$<br>$-21 \le k \le 21$<br>$-28 \le l \le 28$	$-9 \le h \le 9$<br>$-29 \le k \le 32$<br>$-14 \le l \le 14$
Reflections collected	11066	10140
Independent reflections	761 [R_{int} = 0.0290, R_{sigma} = 0.0088]	3544 [R_{int} = 0.0407, R_{sigma} = 0.0474]
Data/restraints/parameters	761/0/49	3544/319/378
Goodness-of-fit on F^2	1.100	1.047
Final *R* indexes [$I \ge 2\sigma(I)$]	R_1 = 0.0368, wR_2 = 0.1066	R_1 = 0.0303, wR_2 = 0.0723
Final *R* indexes [all data]	R_1 = 0.0391, wR_2 = 0.1093	R_1 = 0.0313, wR_2 = 0.0728
Largest diff. peak/hole (e Å^{-3})	0.26/–0.15	1.67/–0.84
Flack parameter	N/A	0.02(1)

Supplementary Table 2. Experimental conditions of the individual 3D-ED datasets of **Pd_3-MOF**.

Measurement No.	Scan range [°]	Scan width [°]	Exposure time/frame [s]	Total exposure time [s]	Dose rate [$e^-/(Å^2 \cdot s)$]	Dose [$e^-/(Å^2)$]
1	–72 to +72	0.25	0.75	432	1.11E–03	0.48
2	–72 to +72	0.25	0.50	288	1.11E–03	0.32
3	–75 to +70	0.25	0.50	290	1.11E–03	0.32
4	–78 to +74	0.25	0.50	304	1.11E–03	0.34
5	–70 to +70	0.25	0.50	280	1.11E–03	0.31

Supplementary Table 3. 3D-ED crystallographic data and refinement parameters for **Pd_3-MOF**.

	**Pd_3-MOF**
CCDC Code	2293025
Empirical formula	$Pd_3(C_{18}H_{16}N_2)_3$
Formula weight	1100.19
Merged Datasets	5
Scan Width (° $frame^{-1}$)	0.25
Temperature (K)	298.15
Crystal system	*Triclinic*
Space group	$P\bar{1}$
a (Å)	8.666(4)
b (Å)	9.401(4)
c (Å)	31.378(10)
α (°)	88.30(3)
β (°)	86.25(3)
γ (°)	72.25(5)
Volume ($Å^3$)	2429.3(18)
Z	2
Resolution (Å)	1.00
ρ_{calc} (g cm^{-3})	1.504
Independent reflections	5063
Completeness (%)	99.4
R_1 (%)	17.9
R_{int} (%)	38.3
R_{pim} (%)	15.1

Supplementary Table 4. Metric data from crystal structures.

	**BXylDI**	**Pd_3**	**Pd_3-MOF**
Pd–Pd (Å)	-	2.66414(2) & 2.71427(3)	2.664(5), 2.672(5), & 2.732(5)
Pd–C (Å):	-	Terminal: 2.009(11) & 2.011(6)<br>Bridging: 2.043(6), 2.075(6), & 2.108(6)	Terminal: 1.9484(11), 1.9699(7), & 2.0054(9)<br>Bridging: 2.0190(7), 2.0338(7), 2.0954(11), 2.1016(12), 2.141(1), & 2.2031(11),
C_{CN}–N (Å):	1.1561(19)	Terminal: 1.145(9) & 1.172(15)<br>Bridging: 1.160(12) & 1.212(8)	Terminal: 1.1493(5), 1.2027(7), & 1.2331(5)<br>Bridging: 1.1784(7), 1.2404(5), & 1.2519(5)
C_{CN}–N–C_{ipso} (°):	180	Terminal: 168.0(6) & 175.1(12)<br>Bridging: 139.9(6) & 146.9(4)	Terminal: 171.957(6), 172.855(5), & 173.005(5)<br>Bridging: 129.83(3), 133.88(3), & 170.638(6)
Pd–C_{bridge}–Pd′ (°):	-	80.2(3) & 80.6(2)	77.94(4), 80.54(4), 80.62(4)
Pd–Pd′–Pd″ (°):	-	59.376(9) & 61.249(18)	59.06(3), 59.33(3), 61.60(3)

Powder X-ray diffraction

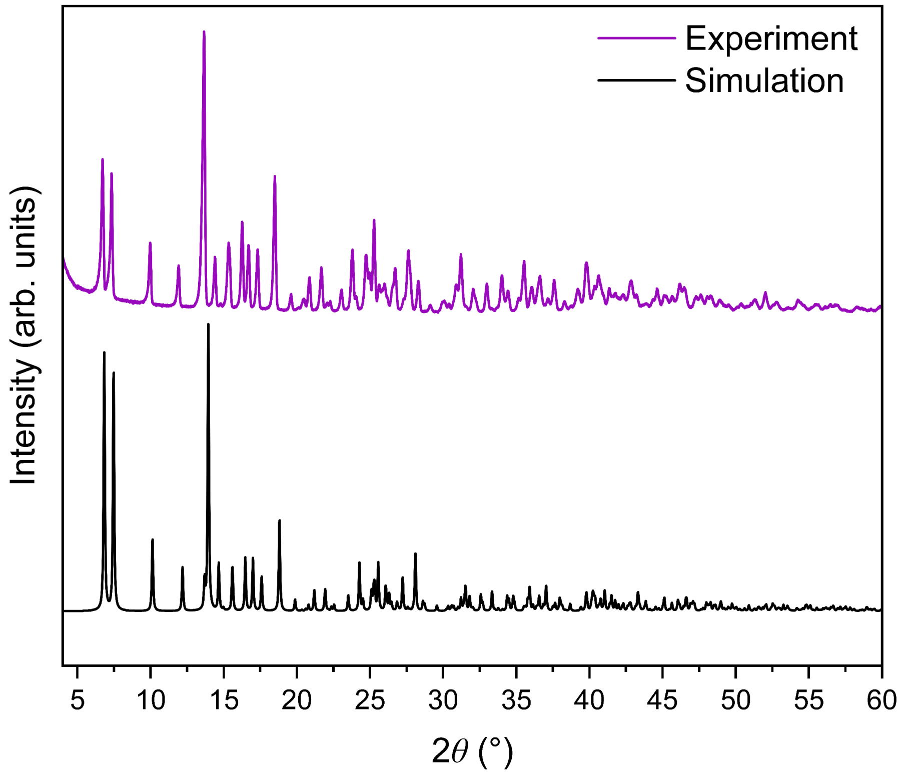

Supplementary Figure 5. Powder X-ray diffractogram (Cu Kα, λ = 1.5406 Å) of **Pd_3** (purple trace) with a simulated diffractogram calculated from the SC-XRD structure (black trace).

Supplementary Note 2

NMR Spectroscopy

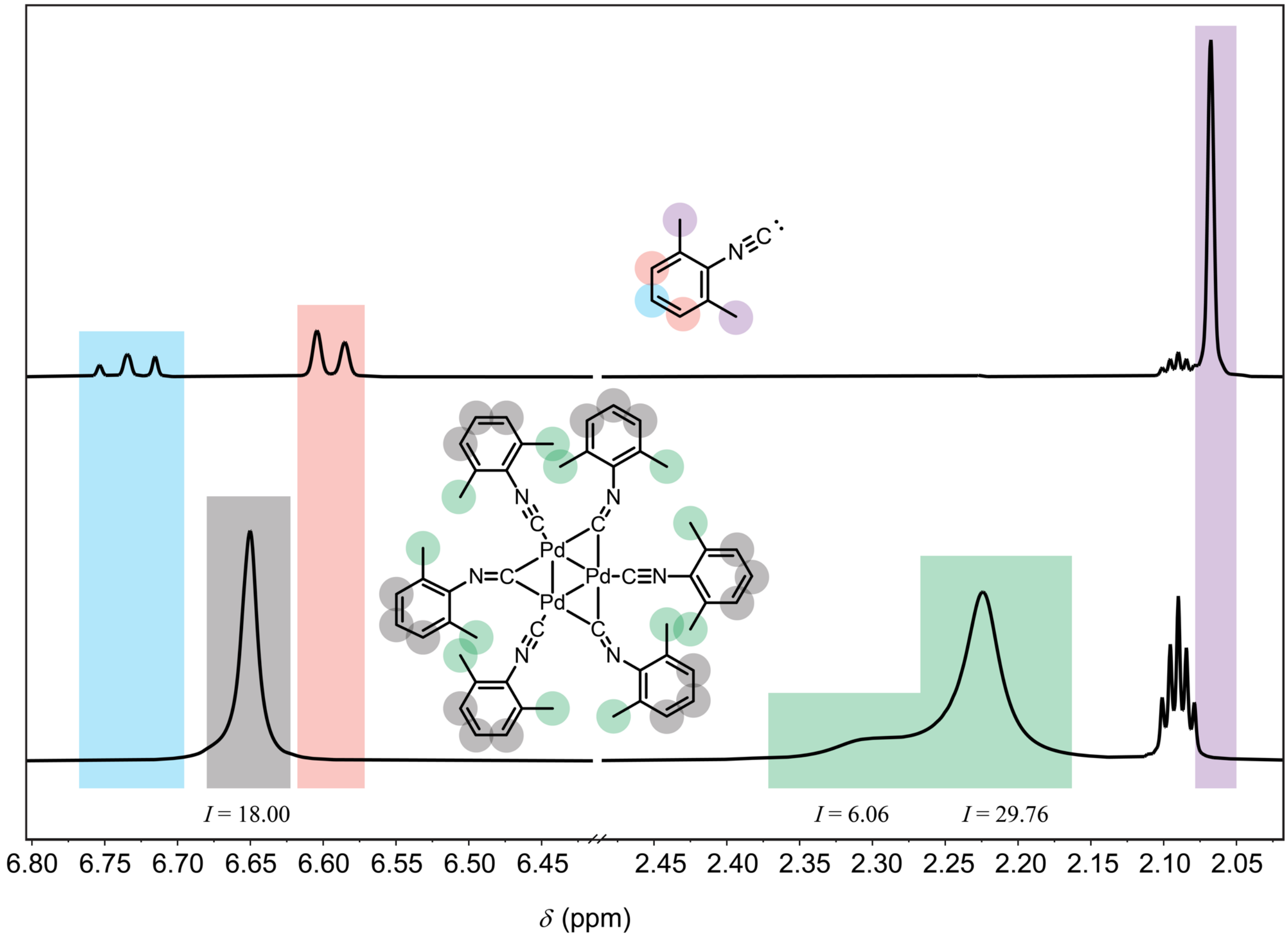

Supplementary Figure 6. ^{1}H-NMR (400 MHz, toluene-d_8, 298 K) spectra of CNXyl (top) and $\mathbf{Pd_3}$ (below).

Supplementary Note 3

UV-vis absorption and diffuse reflectance spectroscopy

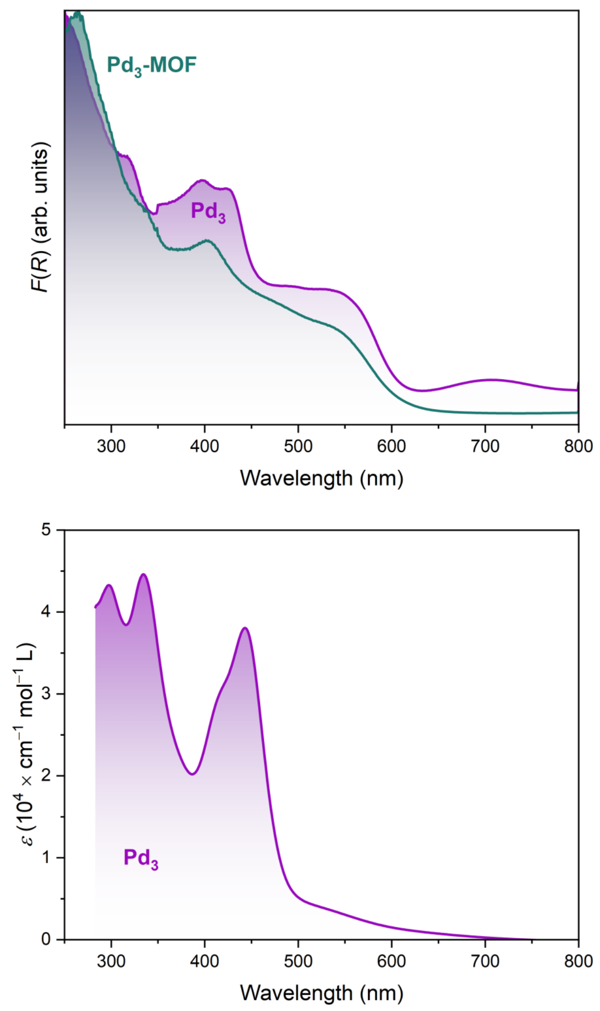

Supplementary Figure 7. Diffuse reflectance spectra of **Pd_3-MOF** and **Pd_3** (top). UV-vis spectrum of **Pd_3** dissolved in toluene (below).

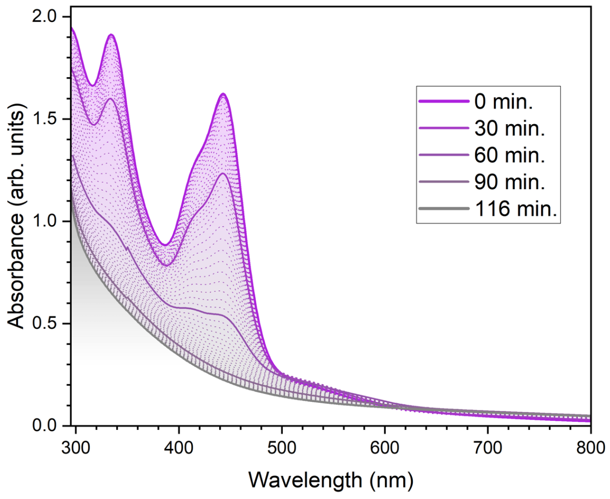

Supplementary Figure 8. UV-vis absorption spectra of **Pd₃** (*ca.* 45 μM) in toluene over time after ambient air was introduced. A spectrum was acquired every two minutes for 116 minutes while the cuvette was open to air, and for clarity, only the spectra collected every thirty minutes are shown with solid lines.

Supplementary Note 4

Infra-Red spectroscopy

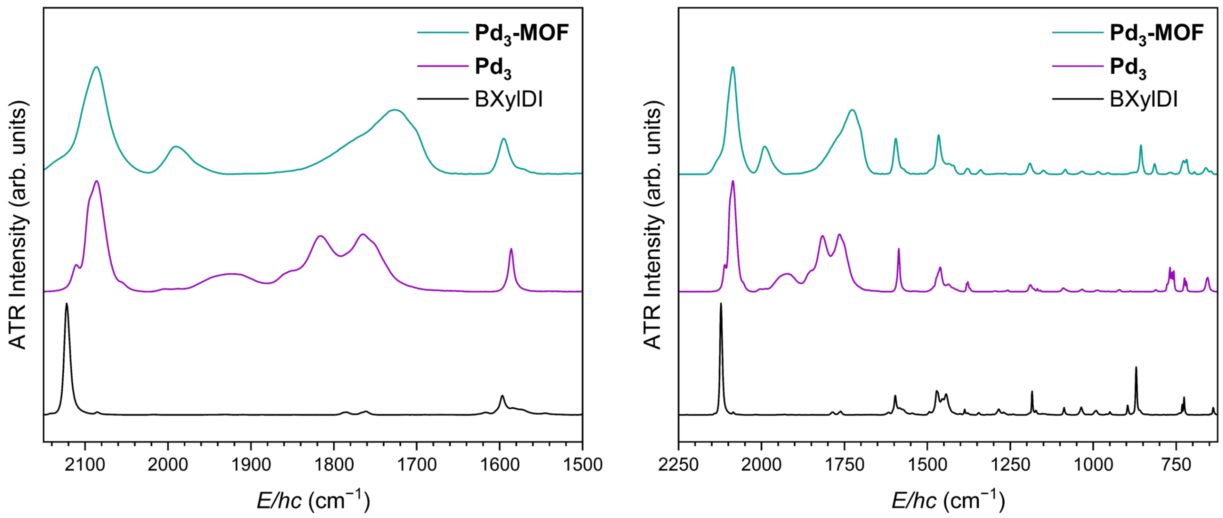

Supplementary Figure 9. ATR-FTIR spectra comparison of the ν(NC) region for **Pd_3-MOF** (green trace), **Pd_3** (purple trace), and BXylDI (black trace, left). Full spectrum (right).

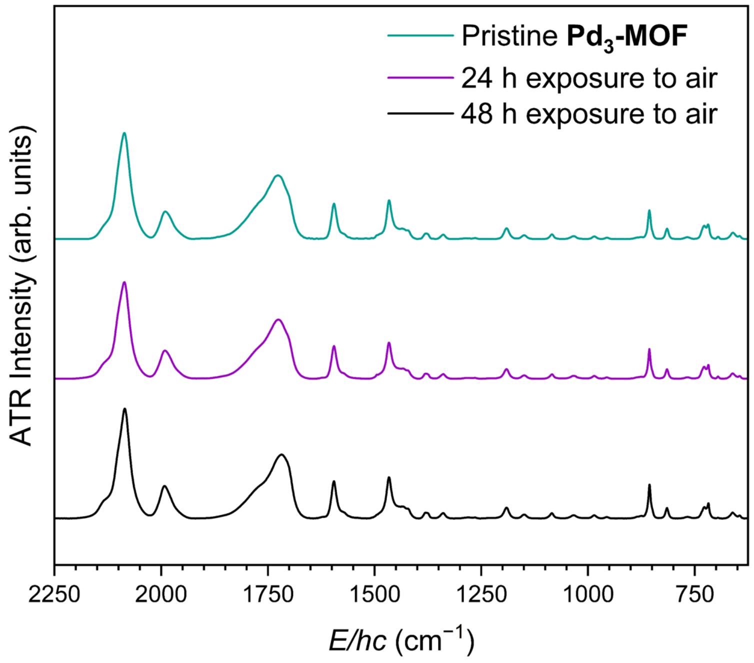

Supplementary Figure 10. ATR-FTIR spectra of pristine **Pd_3-MOF** (green trace), 24 hours after exposure to air (purple trace), and 48 hours after exposure to air (black trace) to monitor the air stability of the MOF. There is essentially no change in the spectra, indicating that the MOF is stable to air for at least 48 hours after exposure to air.

Supplementary Note 5

Gas sorption

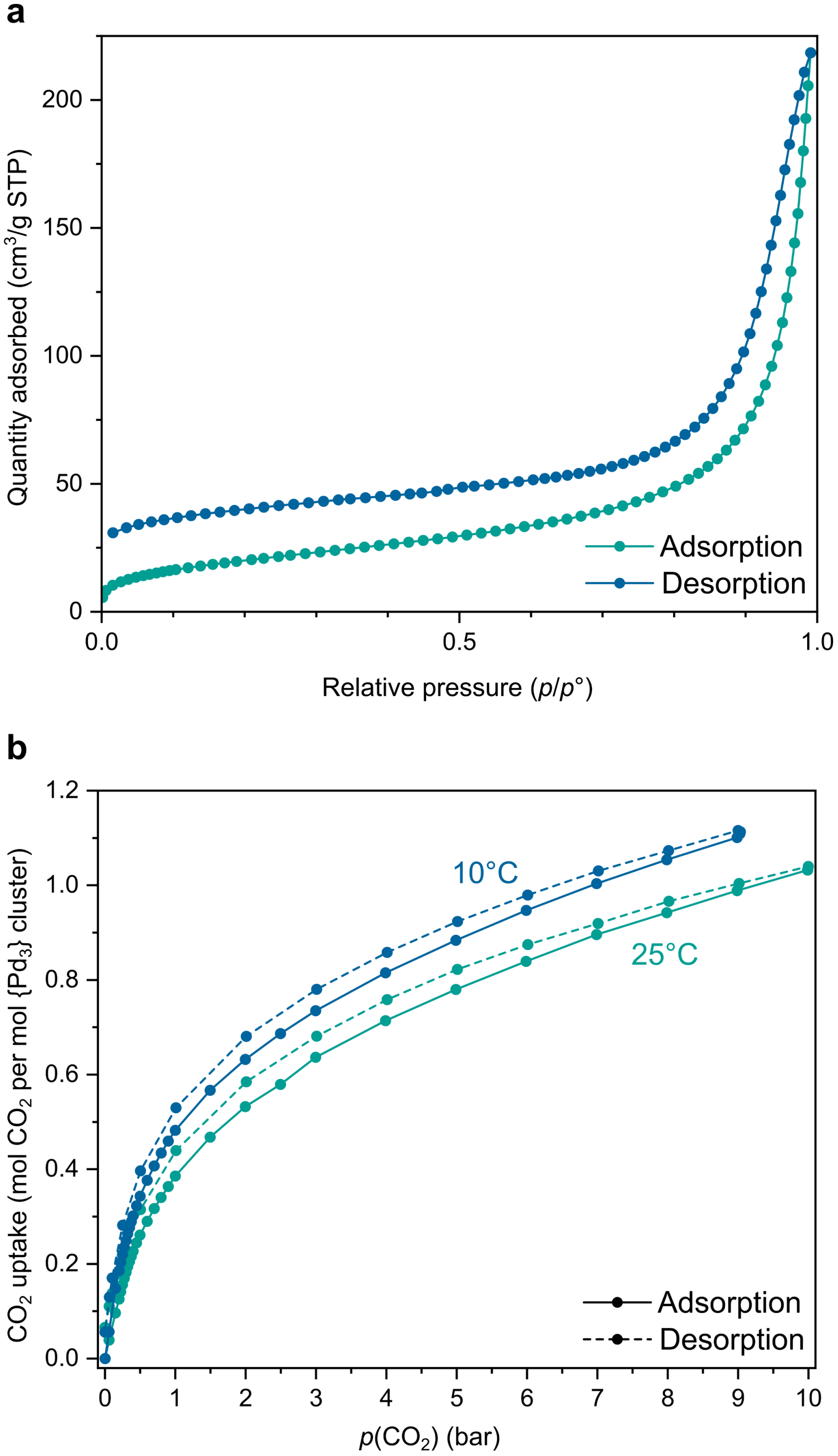

Supplementary Figure 11. a) Volumetric N_2 gas sorption isotherm at −196°C. Adsorption and desorption are plotted in green and blue, respectively. **b)** Gravimetric CO_2 gas sorption isotherms at 25°C (green) and 10°C (blue) for **Pd_3-MOF** up to 9 bar.

Supplementary Note 6

Scanning electron microscopy

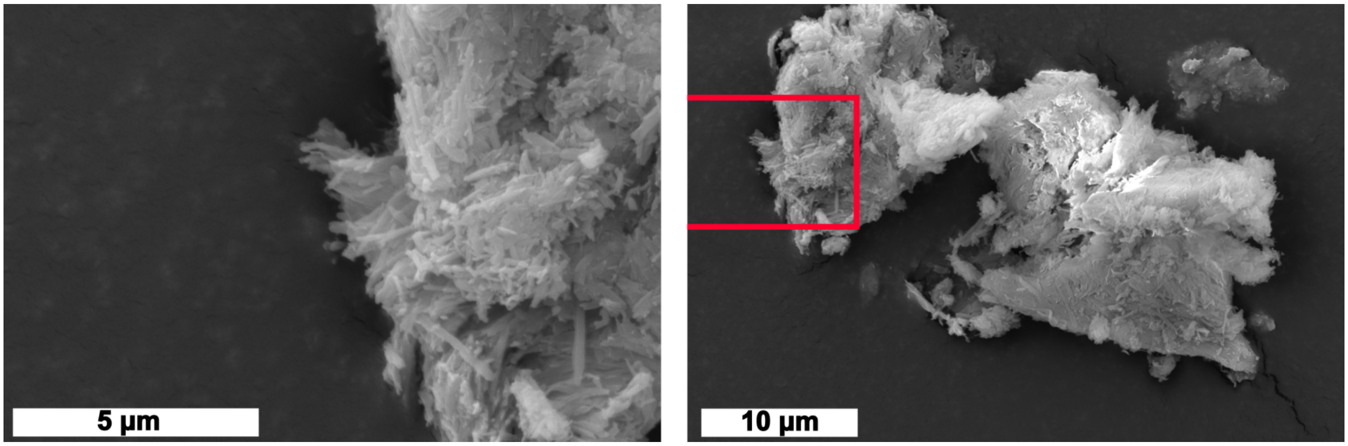

Supplementary Figure 12. SEM images of **Pd_3-MOF**. The area shown in the left image is represented by the red box on the right image.

Supplementary Note 7

X-ray absorption spectroscopy

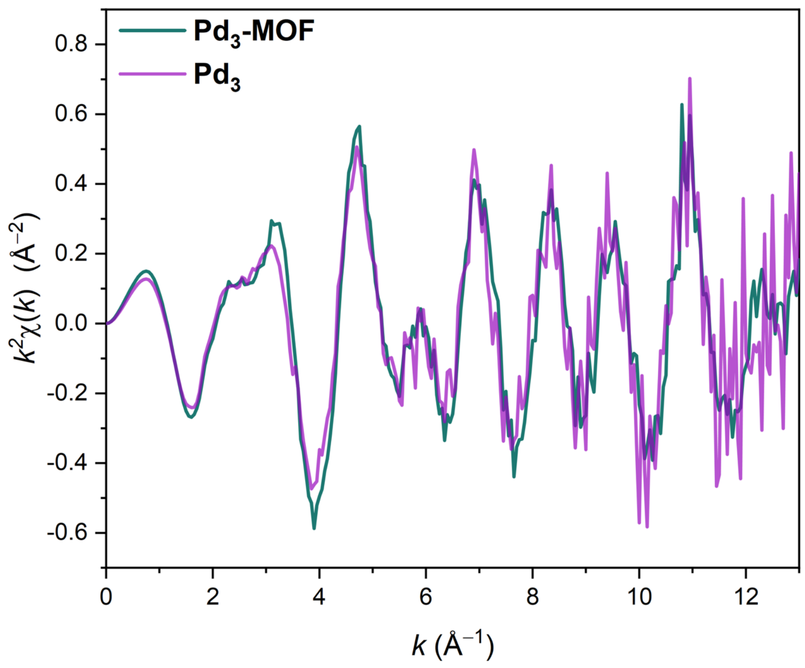

Supplementary Figure 13. Comparison of background corrected and normalized data of **Pd_3-MOF** (green trace) and **Pd_3** (purple trace).

Supplementary Table 5: Paths used in the EXAFS modelling of **Pd_3-MOF**, calculated from FEFF of Pd01.

Scattering path	R_{eff}	Degeneracy
C_{1a}	1.946	1
C_{10C}	2.026	2
Pd_{03}	2.668	2
N_{2C}	3.096	1
N_{2B}	3.145	2
C_{1A}, N_{1A}	3.153	2
C_{1A}, N_{1A}, C_{1A}	3.153	1
C_{10C}, N_{2C}	3.178	2
C_{10B}, N_{2B}	3.210	2
C_{10C}, Pd_{03}	3.271	2

Supplementary Note 8

Thermal stability

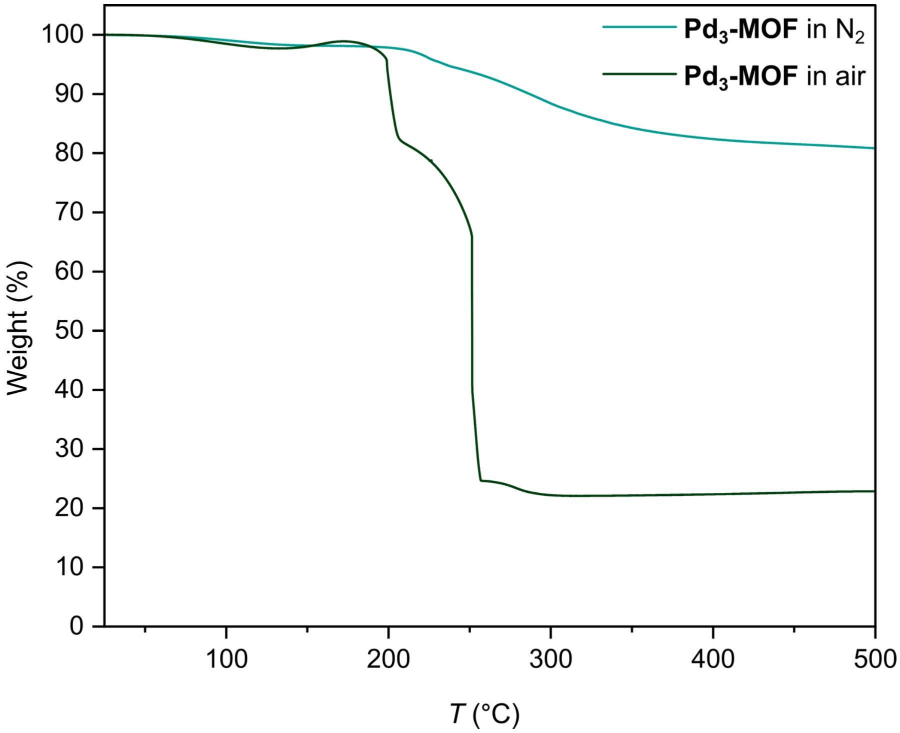

Supplementary Figure 14. TGA of **Pd$_3$-MOF**. Samples were heated at 2 °C min^{-1} from 25 °C to 500 °C under either N_2 (green trace) or ambient (dark green trace) atmospheres.

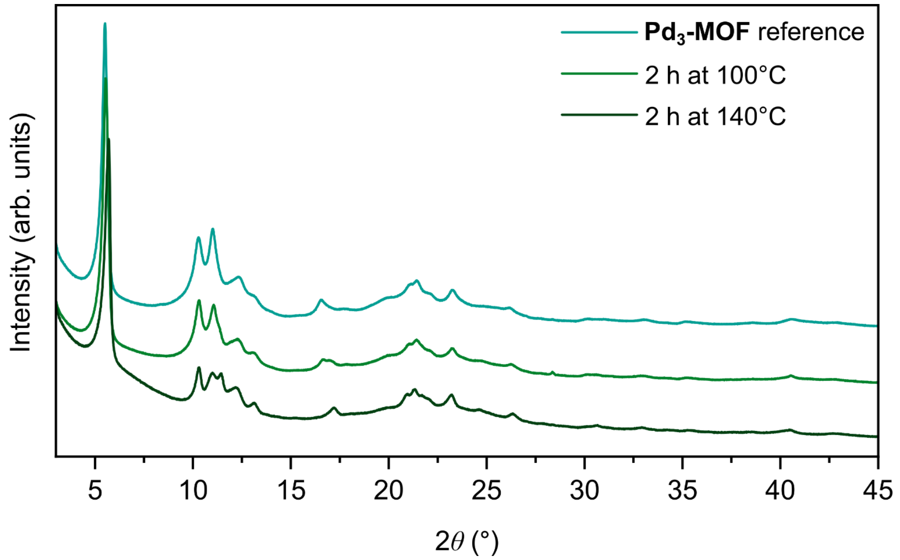

Supplementary Figure 15. Powder X-ray diffractograms (Cu Kα, λ = 1.5408 Å) of **Pd$_3$-MOF** after heating samples in capillaries (0.5 mm diameter, 0.01 mm wall thickness) at 100 °C or 140 °C for 2 hours.

Supplementary Note 9

Solvent stability

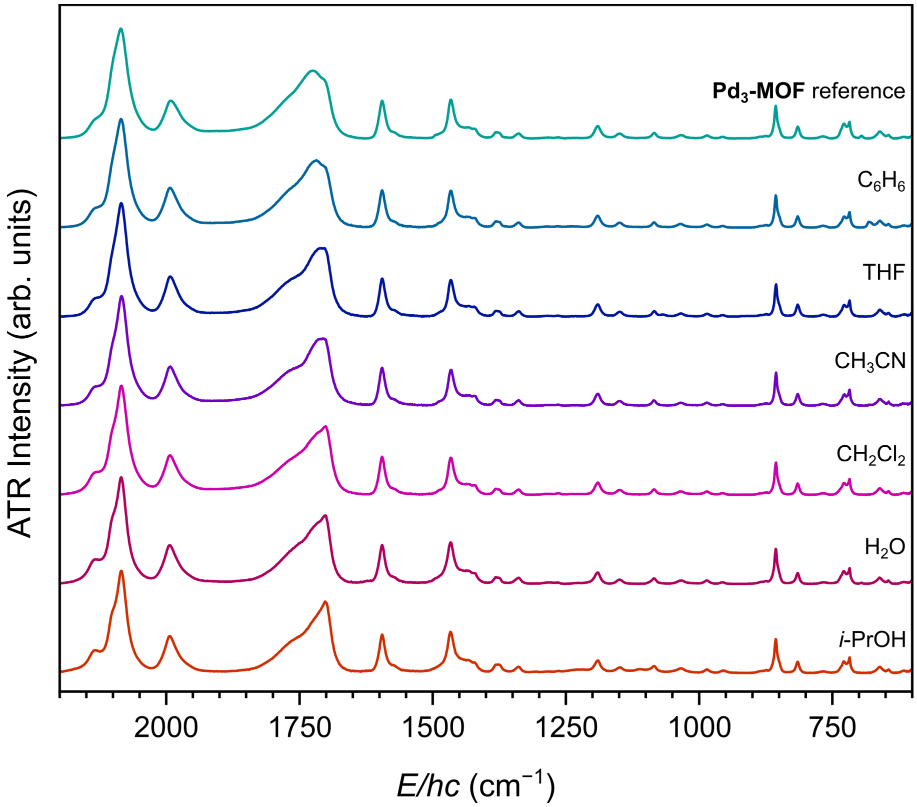

Supplementary Figure 16. ATR-FTIR spectra of dried **Pd_3-MOF** samples after soaking in different solvents for 6 hours.

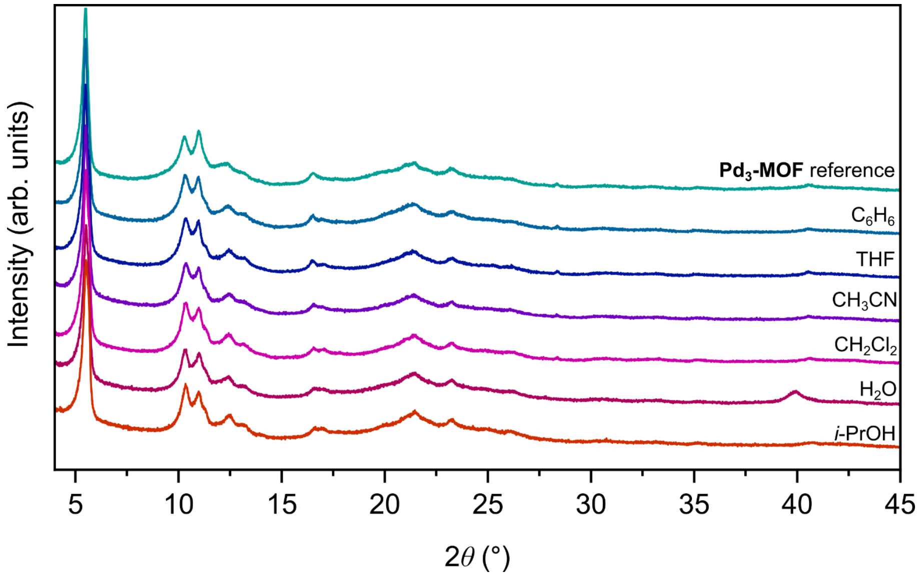

Supplementary Figure 17. Powder X-ray diffractograms (Cu Kα, λ = 1.5408 Å) of dried **Pd_3-MOF** samples after they were soaked in different solvents for 6 hours.

Supplementary Note 10

Reactivity studies

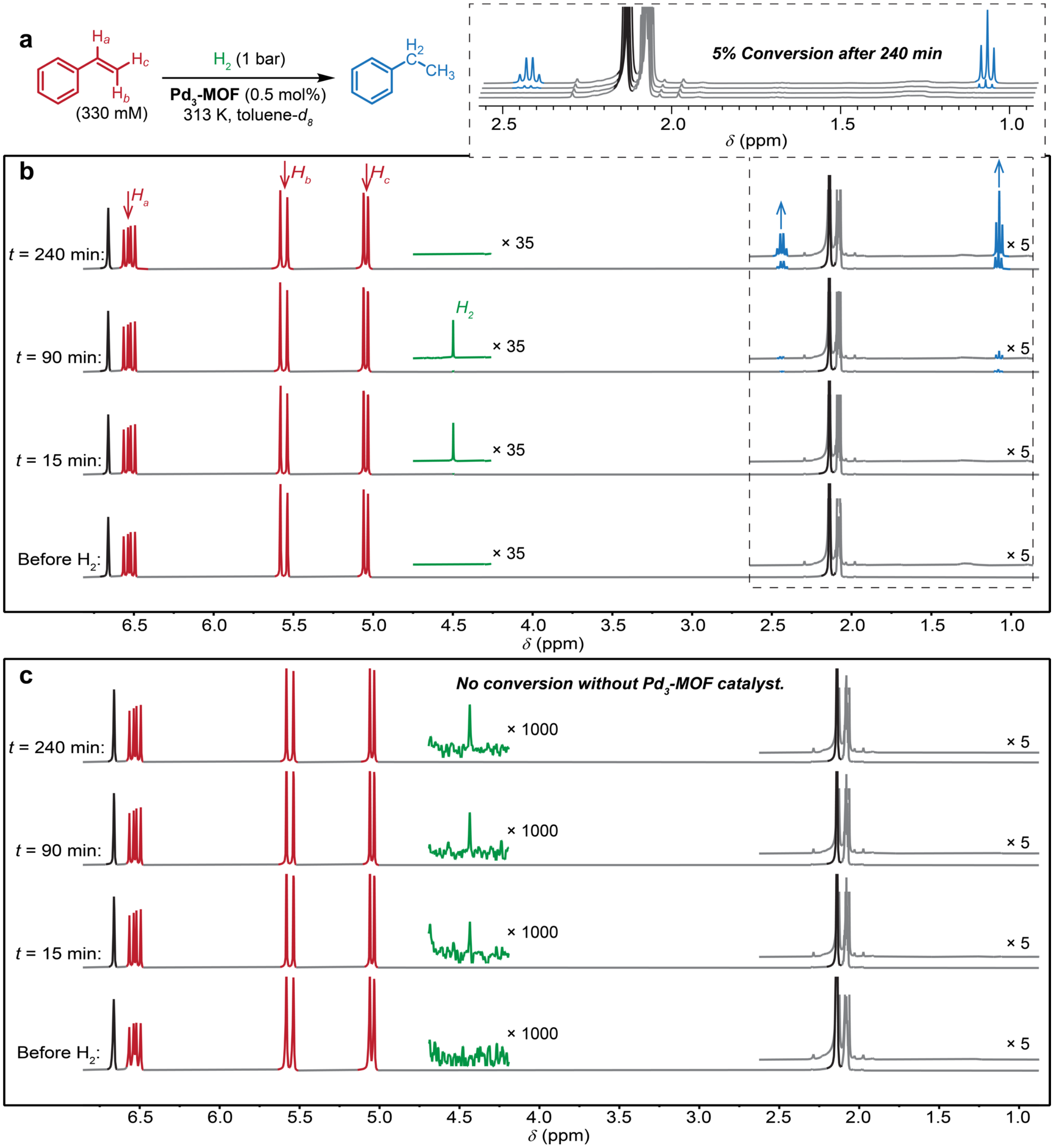

Supplementary Figure 18. a) Catalytic hydrogenation of styrene to ethylbenzene (top). **b** and **c**) ^{1}H NMR (400 MHz, 298 K) spectra of solutions of styrene (330 mM) and mesitylene (55 mM) in toluene-*d*$_8$ acquired before (bottom trace) and at different time intervals after H_2 (1 bar) was introduced to the samples, either in the presence (**b**) or absence (**c**) of **Pd$_3$-MOF** (0.9 mg, 0.8 μmol or 0.5 mol% of "Pd$_3$"). Expansions showing the signals assigned to dissolved H_2 (δ 4.51, green) and ethylbenzene (blue: δ 2.43, *q*, *J* = 7.6 Hz, δ 1.08, *t*, *J* = 7.6 Hz) are shown directly above each spectrum.

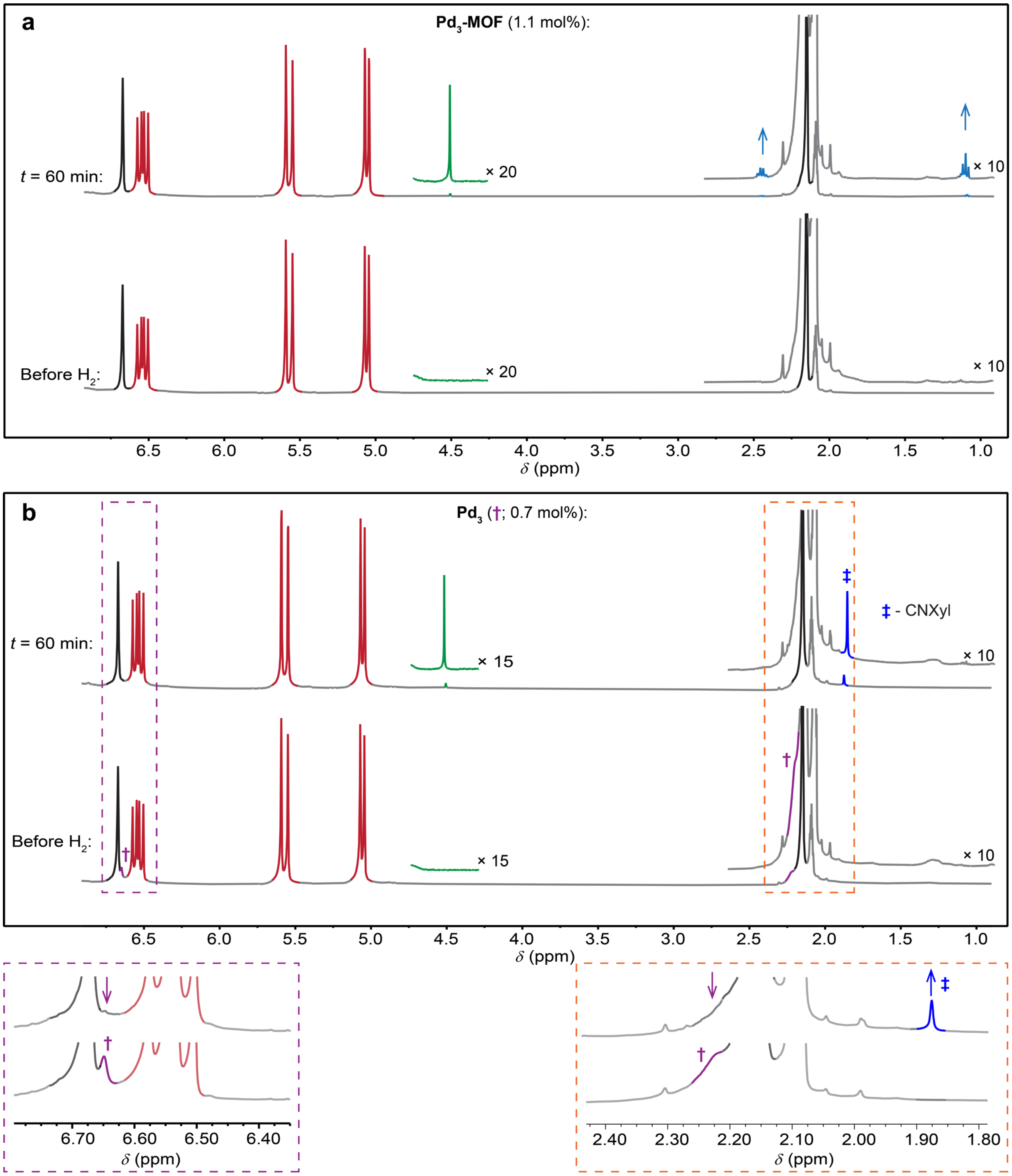

Supplementary Figure 19. ^{1}H NMR (400 MHz, 298 K) spectra of solutions of styrene (330 mM) and mesitylene (55 mM) in toluene-d_8 acquired before (bottom trace) and at different time intervals after H_2 (1 bar) was introduced to the samples, either in the presence **Pd3-MOF** (**a**; 1.6 mg, 1.1 μmol or 0.5 mol% of "Pd3") or **Pd3** (**b**; 1.2 mg, 1.1 μmol, 0.7 mol%). Expansions showing the signals assigned to dissolved H_2 (δ 4.51, green), ethylbenzene (blue: δ 2.43, *q*, *J* = 7.6 Hz, δ 1.08, *t*, *J* = 7.6 Hz) are shown directly above each spectrum, while expansions showing **Pd3** (purple daggers: δ 6.65 and 2.22) and free CNXyl (bright blue double dagger: δ 1.88) are shown below.

Supplementary Table 6: Catalytic hydrogenation of styrene over **Pd_3-MOF** and heterogeneous Pd catalysts.

Catalyst	Loading (mol%)	*P*(H_2) (bar)	Temperature (°C)	Time (min)	Conversion (%)	Ref.
**Pd_3-MOF**	0.5[a]	1	40	240	5	-
-	-	1	40	240	0	-
**Pd_3-MOF**	1.1[a]	1	40	60	0.9	-
**Pd_3**	0.7	1	40	60	0	-
Pd/C	0.1	-	25	20	46 ± 12	[1]
Pd/WMC	0.1	-	25	20	68 ± 2	[1]
Fe_3O_4dpa@$Pd_{0.5}$	2 wt%	3	25	60	-	[2]
Pd/Tm-MOF	1 wt%	1	35	90	10.3	[3]
Pd/ZIF-8	1 wt%	1	35	90	4.5	[3]
Pd/MOF-5	1 wt%	1	35	90	10	[4]
Pd complex/polymer	0.2	1	25	234	98	[5]
$(PdCl_2/bpy)_{10}$	-	1	25	40	91	[6]
L@PdNP	0.1 wt%	10	25	16 h	100	[7]
Pd@CuBTC-C_{10}ip MOF	0.06	1	25	30	72	[8]
PdNP	2.5 wt%	1	40	16 h	99	[9]
Pd/C	2.5 wt%	1	40	16 h	99	[9]
PdNP@organosilica	0.15	1	25	24 h	99	[10]

[a]calculated per mol of “Pd_3”.

Supplementary References

1. Wang, Z., Balkus J. & K. J. Wrinkled mesoporous carbon supported Pd nanoparticles for hydrogenation and aerobic oxidation reactions. *J. Porous Mater.*, **25**, 15–21 (2018).
2. Guarnizo, A. *et al.* Highly water-dispersible magnetite-supported Pd nanoparticles and single atoms as excellent catalysts for Suzuki and hydrogenation reactions. *RSC Adv.*, **6**, 68675–68684 (2016).
3. Pan, Y. *et al.* Uncoordinated carbonyl groups of MOFs as anchoring sites for the preparation of highly active Pd nano-catalysts. *J. Mater. Chem.*, **22**, 10834–10839 (2012).
4. Sabo, M., Henschel, A., Fröde, H., Klemm, E. & Kaskel, S. Solution infiltration of palladium into MOF-5: synthesis, physisorption and catalytic properties. *J. Mater. Chem.*, **17**, 3827–3832 (2007).
5. Islam, S. M., Roy, A. S., Mondal, P. & Salam, N. Selective hydrogenation and Suzuki cross-coupling reactions of various organic substrates using a reusable polymer-anchored palladium(II) Schiff base complex. *Appl. Organomet. Chem.*, **26**, 625–634 (2012).
6. Gao, S., Li, W. & Cao, R. Palladium-pyridyl catalytic films: A highly active and recyclable catalyst for hydrogenation of styrene under mild conditions. *J. Colloid Interface Sci.*, **441**, 85–89 (2015).
7. Ferry, A. *et al.* Negatively charged N-heterocyclic carbene-stabilized Pd and Au nanoparticles and efficient catalysis in water. *ACS Catal.*, **5**, 5414-5420 (2015).
8. Fan, Z. *et al.* Enhanced catalytic performance of palladium nanoparticles in MOFs by channel engineering. *Cell Rep. Phys. Sci.*, **3**, 100757 (2022).
9. Rühling, A. *et al.* Modular bidentate hybrid NHC-thioether ligands for the stsabilization of palladium nanoparticles in various solvents. *Angew. Chem. Int. Ed.*, **55**, 5856 - 5860 (2016).
10. Wang, L. *et al.* Covalent immobilization of imidazolium cations inside a silica support: Palladium-catalyzed olefin hydrogenation. *ChemCatChem*, **4**, 395 - 400 (2012).
